# Cesarean delivery on child health and development in Japanese nationwide birth cohort

**DOI:** 10.1038/s41598-025-87043-2

**Published:** 2025-01-20

**Authors:** Naomi Matsumoto, Takashi Mitsui, Kei Tamai, Tomoya Hirota, Hisashi Masuyama, Takashi Yorifuji

**Affiliations:** 1 Department of Epidemiology, Faculty of Medicine, Dentistry and Pharmaceutical Sciences, Okayama University, 2-5-1 Shikata-cho, Kita- ku, Okayama, Japan; 2 Department of Obstetrics and Gynecology, Okayama University Graduate School of Medicine, Okayama, Japan; 3 Division of Neonatology, NHO Okayama Medical Center, Okayama, Japan; 4 Department of Psychiatry and Behavioral Sciences, UCSF Weill Institute for Neurosciences, University of California San Francisco, San Francisco, USA

**Keywords:** Cesarean delivery, Delivery methods, Long-term outcome, Child development, Outcome-wide approach, Epidemiology, Outcomes research, Paediatric research

## Abstract

The long-term effects of cesarean delivery (CD) on child health and development remain controversial. This study aimed to investigate these effects using an outcome-wide approach in a Japanese context, where perinatal mortality rates are among the world’s lowest. We analyzed data from 2,114 children in a nationwide Japanese birth cohort, linking the 21st Century Longitudinal Survey of Newborns with the Perinatal Research Network database. We examined associations between CD and various health and developmental outcomes up to 9 years of age, including hospitalizations, obesity, and developmental milestones. After adjusting for potential confounders, CD was not significantly associated with most outcomes, including all-cause hospitalization (adjusted risk ratio 1.25, 95% CI 0.997–1.56), obesity at 5.5 and 9 years, and various developmental milestones. Subgroup analyses for multiple births and preterm infants showed some differences in point estimates, but were limited by small sample sizes. CD was not significantly associated with adverse long-term child health or developmental outcomes in this Japanese cohort. These findings provide reassurance regarding CD safety when medically indicated in advanced perinatal care settings. Further research with larger samples and longer follow-up is needed, especially for specific subgroups.

**Supplementary Information:**

The online version contains supplementary material available at 10.1038/s41598-025-87043-2.

## Introduction

Mode of delivery has the potential to affect a child’s health and development in the long term^1^. Cesarean delivery (CD) is often performed for certain medical indications to ensure the safety of both the mother and the child^2,3^. Although CD rates have risen dramatically worldwide over the past decades, the potential consequences of this surgical intervention on various aspects such as physical growth, cognitive development, and risk of chronic diseases in children are the subject of ongoing debate.

CDs often result from pregnancy complications or maternal health conditions rather than being a primary cause of adverse outcomes. Factors such as gestational diabetes, preeclampsia, or fetal distress may necessitate CD and independently influence child health outcomes^4–6^. However, biological mechanisms exist through which CD itself may affect child health and developmental outcomes. For instance, CD may alter infant’s microbiome colonization, as these infants bypass exposure to maternal vaginal and intestinal flora during birth^7^. This altered microbial colonization could potentially affect immune system development^8^, metabolic processes, and even neurodevelopment^9–11^. Previous studies have reported mixed findings regarding the effects of CD on long-term health and developmental outcomes^12–14^. Some studies have suggested potential benefits of CD, such as lower morbidity and better neurodevelopmental outcomes in extremely low-birth-weight infants^3^However, other investigations have raised concerns about potential negative outcomes^12,15^. For example^16^, some studies have reported a higher body mass index (BMI) at grade 6 in infants born via CD^17^.

These conflicting findings highlight the complexity of the issue and the need for further research, particularly in specific cultural and health care contexts. In Japan, where the CD rate is approximately 20%, the Japan Environment and Children’s Study (JECS) found no significant relationship between CD and infectious diseases in one-year-old children^18^, contrasting with several international studies^19–21^. This discrepancy underscores the potential influence of country-specific factors on outcomes. Additionally, while the Japan Environment and Children’s Study (JECS) suggested a potential association between CD and the prevalence of autism at age 3^22^, further investigation is needed regarding the relationship between CD and neurodevelopmental disorders or long-term cognitive development in Japanese children, particularly beyond early childhood.

In this study, we utilized the 21st Century Longitudinal Survey of Newborns, a nationally representative birth cohort linked to the Perinatal Research Network (PRN) database, to investigate the relationship between CD and various child health and developmental outcomes. By focusing on outcomes from 1.5 to 9 years of age, we extend beyond the early childhood period typically studied, providing insights into the potential persistent effects of CD into school age. Moreover, we adopted an outcome-wide approach, encompassing both physical health outcomes (such as hospitalizations for various diseases and obesity) and neurodevelopmental outcomes (including developmental, behavioral, and cognitive issues). This comprehensive approach allows for a more holistic understanding of how CD may impact diverse aspects of children’s physical and mental health, potentially revealing patterns and associations that might be overlooked in studies focusing on single outcomes^23,24^.

## Methods

### Study design/setting/participants

This population-based cohort study leveraged two nationally representative data sources that provide a foundation for investigating child health outcomes in Japan: the 21st Century Longitudinal Survey of Newborns and the Perinatal Research Network (PRN) database.

The 21st Century Longitudinal Survey of Newborns, conducted by the Ministry of Health, Labour, and Welfare of Japan, is a national birth cohort that encompasses all 43,767 infants born in the country between May 10 and 24, 2010^25^. This cohort represents one twenty-fourth of all births in Japan in 2010, ensuring representativeness of the entire birth population. Questionnaires were sent annually to the guardians of 38,554 children (response rate: 88.1%) who responded to an initial questionnaire at 6 months of age. The survey continued until age 5.5 years, with additional follow-ups at ages 7, 8, and 9 years. This longitudinal design allows for comprehensive tracking of child development and health outcomes over time. The questionnaires covered a wide range of topics including the child’s physical development^26,27^, medical history^28,29^, parental employment and educational attainment, parental smoking history, and parenting concerns. Additionally, official birth-related data from the Japanese Vital Statistics were linked to each participant, further enhancing the dataset’s accuracy.

The Perinatal Research Network (PRN) database was initiated by the Japan Society of Obstetrics and Gynecology in the early 2000s, and serves as a nationwide registry of births and stillbirths after 22 weeks of gestation. The participating facilities were predominantly maternal and child medical centers managing complex cases, providing crucial insights into perinatal care for at-risk populations. The PRN contains detailed clinical information recorded by obstetricians on maternal characteristics, preexisting medical conditions, pregnancy complications, delivery details, neonatal transport, and other relevant factors. In 2010, the PRN registered data on 83,383 births from 139 facilities, representing 7.6% of all births in Japan that year^30,31^. The cesarean delivery rate in this database for May 2010 births was 33.4%, higher than the national rate of 19.2% reported in September 2011^32^, reflecting its emphasis on high-risk pregnancies and deliveries.

**Fig. 1:**
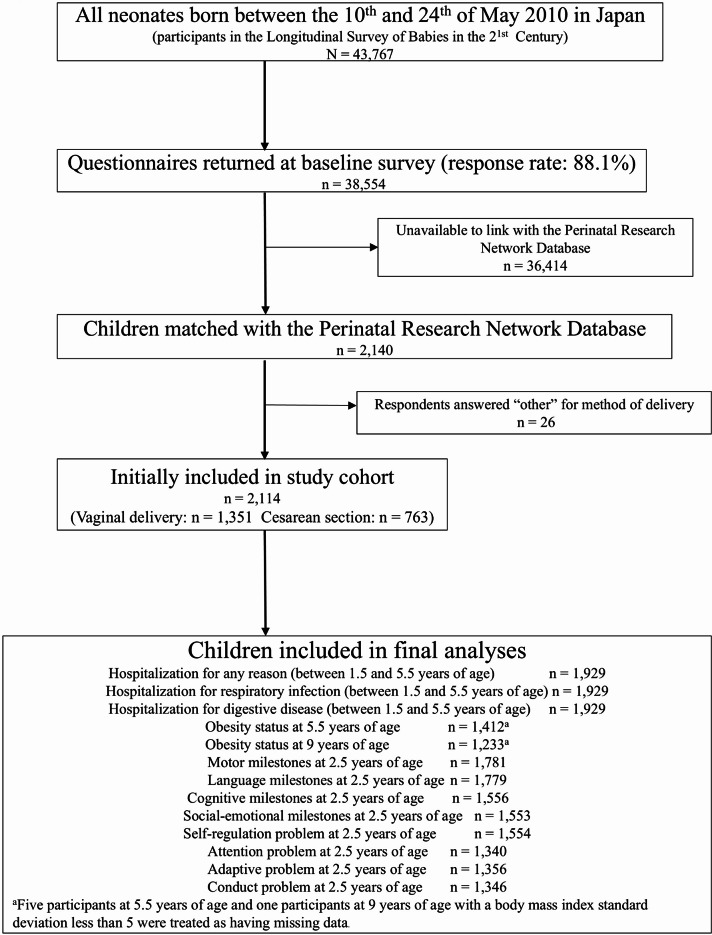
Flowchart of the participants.

By linking these two nationally representative data sources using information on birth date, sex, birth weight, maternal age at birth, and gestational age, we created a unique dataset that combines the strengths of a nationally representative birth cohort with detailed perinatal clinical data. Of the 2,140 infants whose data were successfully linked, 2,114 were included in our final analysis after excluding 26 children with ambiguous delivery method responses (e.g., “other”). This integrated dataset provides a valuable opportunity to examine the long-term effects of perinatal factors on child health and development in Japan (Fig. 1).

## Exposure and outcomes

The primary exposure was the mode of delivery (cesarean vs. vaginal), as recorded in the PRN database. We examined multiple outcomes from ages 0.5 to 9 years through the surveys conducted at ages 1.5 to 9 years. Hospitalizations (any hospitalization and specific hospitalizations for respiratory infections and gastrointestinal illnesses) were defined as admission experience between ages 0.5 and 5.5 years, as reported in surveys at ages 1.5 to 5.5 years. Obesity was assessed at ages 5.5 and 9 years. Body mass index (BMI) standard deviation scores were calculated based on WHO criteria. Developmental milestones (including motor, language, cognitive, self-regulation, social-emotional, attention, adaptive skills, and conduct problems) were evaluated at ages 2.5, 5.5, and 8 years.

Detailed definitions of these outcomes are available in eMethod and Table S1.

### Statistical analysis

The baseline characteristics of parents and newborns were compared between CD and vaginal delivery groups. To assess potential selection bias due to loss to follow-up, we compared characteristics between the dropout and non-dropout (analytic sample) groups, focusing primarily on the longest-term outcome: obesity at 9 years of age. Categorical variables were summarized as frequencies (percentages), and continuous variables were described using the median (Interquartile Range [IQR]) and minimum and maximum values.

Poisson regression with robust variance was then used to estimate risk ratios for the association between CD and various long-term child health and development outcomes, including hospitalization, obesity, and developmental milestones. Following the crude analysis (Model 1), a controlled analysis (Model 2: complete case analysis) was conducted to adjust for potential confounding variables. These covariates were selected based on prior research and subject-matter knowledge^33,34^: multiple births (binary), fetal presentation (cephalic, non-cephalic; binary), presence of fetal anomalies (binary), maternal transport (none, emergency, scheduled; categorical), maternal age at delivery (< 30 years, 30–34 years, ≥ 35 years; categorical), multiparity (binary), pre-existing maternal medical conditions (binary), pregnancy complications (binary), maternal smoking during pregnancy (binary), maternal alcohol consumption during pregnancy (binary), maternal educational attainment (bachelor’s degree or higher, technical/associate’s degree, high school diploma or less; categorical), paternal age at delivery (categorized analogous to maternal age), paternal educational attainment (categorized analogous to maternal education), and place of residence at birth (special ward or designated city, city, and town or village; categorical). Pre-pregnancy maternal BMI (continuous) was included as a covariate in the obesity outcome model. In Model 3, multiple imputation was used to replace missing covariate data using the chained equations method under the Missing At Random assumption^35^. The proportion of missing data ranged from 0 to 25.7%. Multiple imputation was performed with 400 iterations and 20 burn-ins and incorporated the following variables: multiple births (no missing data), fetal presentation (no missing data), fetal anomalies (no missing data), gestational age (no missing data), maternal transport (no missing data), maternal age (no missing data), multiparity (missing data: 0.7%), pre-pregnancy maternal BMI (missing data: 15.8%), maternal smoking (missing data: 25.5%), maternal alcohol consumption (missing data: 25.7%), pre-existing maternal conditions (no missing data), pregnancy complications (no missing data), maternal education (missing data: 12.6%), paternal age (missing data: 2.5%), paternal education (missing data: 14.0%), and place of residence at birth (no missing data). E-values were calculated for each association to assess the potential impact of unmeasurable confounding; a higher E value indicates that stronger unmeasured confounding is required to shift the observed association to null^36,37^.

For the primary analysis, we compared the CD group (combining both scheduled and emergency cesareans) with the vaginal delivery group. Additionally, we conducted supplementary analyses to examine potential differences between delivery methods. In these analyses, we separated the CD group into “scheduled cesarean” and “emergency cesarean” subgroups, allowing for a more nuanced comparison of outcomes across different delivery methods. In addition, subgroup analyses were performed stratified by the presence or absence of multiple births and preterm birth.

All analyses were conducted using STATA SE version 18 (StataCorp., College Station, TX, USA). For all analyses, we initially set the significant level at 5% (*p*< 0.05). For results that were initially significant, we applied Bonferroni correction to account for multiple testing^38^. This study was approved by the Institutional Review Board of the Graduate School of Biomedical Sciences at Okayama University (No. 2310-018) and the Clinical Research Management Review Committee of the Japanese Society of Obstetrics and Gynecology (No. 150). The Institutional Review Board of the Graduate School of Biomedical Sciences at Okayama University waved the requirement for informed consent due to the retrospective nature of the study and because the datasets were fully anonymized for secondary use. All methods were performed in accordance with the principles of the Declaration of Helsinki and the relevant guidelines and regulations.

## Results

Table 1 presents baseline characteristics stratified by mode of delivery. Compared with infants born by vaginal delivery, those delivered by CD had higher proportions of older parents, multiparous mothers, preterm birth, low birth weight, small size for gestational age, multiple gestation, prenatal fetal anomalies, neonatal asphyxia, maternal emergency transport, in-vitro fertilization, and breech presentation. Although pre-existing maternal conditions and pregnancy complications were more prevalent in the CD group, maternal alcohol consumption during pregnancy was lower than in the vaginal delivery group. The CD group showed a slightly lower proportion of children residing in designated special wards or cities, and conversely, a higher proportion living in cities, towns, or villages compared to the vaginal delivery group. To evaluate potential selection bias, demographic characteristics were compared between the dropout and non-dropout groups at the 9-year follow-up (Table S2). The parents in the dropout group were younger, had lower educational attainment, and had a higher prevalence of maternal smoking during pregnancy compared with those in the non-dropout group.The detailed numbers and percentages of the evaluated outcomes are shown in Table S3.

**Table 1:** Demographics of participants.

	Mode of delivery		
	Vaginal delivery	Cesarean section	All
	(*n* = 1,351)	(*n* = 763)	(*N* = 2,114)
Gestational week^a^	39 (38–40)	37 (36–38)	38 (37–40)
Preterm birth	119 (8.8%)	196 (25.7%)	315 (14.9%)
Birth weight (g) ^a^	2,988 (2,710-3,240)	2,670 (2,255-3,005)	2,891.5 (2,546-3,168)
Low birth weight < 2500 g	192 (14.2%)	279 (36.6%)	471 (22.3%)
Small for gestational age	50 (3.7%)	71 (9.3%)	121 (5.7%)
Multiple births	20 (1.5%)	129 (16.9%)	149 (7.0%)
Neonatal asphyxia			
No	1,317 (98.1%)	717 (94.6%)	2,034 (96.8%)
Mild neonatal asphyxia	24 (1.8%)	35 (4.6%)	59 (2.8%)
Severe neonatal asphyxia	2 (0.1%)	6 (0.8%)	8 (0.4%)
Maternal transfer			
No	1,174 (86.9%)	610 (79.9%)	1,784 (84.4%)
Yes (emergent)	61 (4.5%)	73 (9.6%)	134 (6.3%)
Yes (non-urgent)	116 (8.6%)	80 (10.5%)	196 (9.3%)
Parity			
Primipara	755 (56.3%)	357 (47.0%)	1,112 (53.0%)
Multipara	585 (43.7%)	403 (53.0%)	988 (47.0%)
Maternal underlying conditions	385 (28.5%)	275 (36.0%)	660 (31.2%)
*in-*vitro fertilization	52 (3.8%)	64 (8.4%)	116 (5.5%)
Position			
Cephalic presentation	1,347 (99.7%)	608 (79.7%)	1,955 (92.5%)
Breech presentation or others	4 (0.3%)	155 (20.3%)	159 (7.5%)
Pregnancy complications	733 (54.3%)	497 (65.1%)	1,230 (58.2%)
Prenatal fetal anomalies	87 (6.4%)	76 (10.0%)	163 (7.7%)
Maternal smoking during pregnancy	50 (5.0%)	31 (5.3%)	81 (5.1%)
Maternal alcohol consumption during pregnancy	49 (5.0%)	12 (2.1%)	61 (3.9%)
Maternal age at delivery			
<30	454 (33.6%)	180 (23.6%)	634 (30.0%)
30 ~ 34	476 (35.2%)	274 (35.9%)	750 (35.5%)
35~	421 (31.2%)	309 (40.5%)	730 (34.5%)
Paternal age at delivery			
<30	337 (25.5%)	136 (18.3%)	473 (22.9%)
30 ~ 34	410 (31.1%)	215 (28.9%)	625 (30.3%)
35~	572 (43.4%)	392 (52.8%)	964 (46.8%)
Maternal education attainment			
Bachelor’s degree or higher	368 (31.3%)	197 (29.4%)	565 (30.6%)
Vocational school/junior college graduate	495 (42.1%)	296 (44.2%)	791 (42.8%)
High school graduate or below	314 (26.7%)	177 (26.4%)	491 (26.6%)
Paternal education attainment			
Bachelor’s degree or higher	623 (53.8%)	347 (52.7%)	970 (53.4%)
Vocational school/junior college graduate	185 (16.0%)	104 (15.8%)	289 (15.9%)
High school graduate or below	350 (30.2%)	208 (31.6%)	558 (30.7%)
Place of residence at birth			
Special ward or designated city	615 (45.5%)	293 (38.4%)	908 (43.0%)
City	661 (48.9%)	420 (55.0%)	1,081 (51.1%)
Town or village	75 (5.6%)	50 (6.6%)	125 (5.9%)

Categorical variables were described by number (%), and continuous variables^a^ were displayed as Median (Interquartile Range [IQR]).

Range (minimum-maximum values) for continuous variables:.

Gestational week: All (22–42), Vaginal delivery (22–42), Cesarean Sects. (22–42).

Birth weight: All (486-4,934 g), Vaginal delivery (524-4,446 g), Cesarean Sects. (486-4,934 g).

No statistically significant associations with CD were observed in almost all health and developmental outcomes in children after adjusting for potential confounders. The association between CD and adaptive problems at 8 years of age was initially significant (*p* = 0.02), but not after Bonferroni correction for 13 comparisons (adjusted *p* = 0.26). The analysis showed slightly higher point estimates for risks for some hospitalization outcomes in children born by CD, although these differences did not reach statistical significance: all-cause hospitalization, (aRR 1.25, 95% CI 0.997–1.56), hospitalization due to respiratory infections (aRR 1.29, 95% CI 0.89–1.89) and gastrointestinal diseases (aRR 1.13, 95% CI 0.56–2.31). CD showed no significant association with overweight or obesity at 5.5 years (aRR 1.05, 95% CI 0.68–1.62) or 9 years of age (aRR 0.83, 95% CI 0.52–1.32). Regarding developmental outcomes, CD was not significantly associated with motor, language, cognitive, and social-emotional milestone attainment by the expected time. Similarly, no significant associations were found for self-regulation problems at 5.5 years, or attention and conduct problems at 8 years. The results were consistent across the imputed and non-imputed models (Table 2).

**Table 2:** Cesarean Delivery Effects on Child Outcomes

	Cesarean delivery	Vaginal delivery	Crude model				Adjusted model				Multiple imputation adjusted model^a^			E-value
	n/N (%)	n/N (%)	RR	95%CI		E-value	aRR	95%CI		E-value	aRR	95%CI		
*Health outcomes*														
Hospitalization for any reason	186/698 (26.7%)	267/1,231 (21.7%)	1.23	1.04	1.45	1.76	1.25	0.997	1.56	1.81	1.19	0.99	1.43	1.67
Hospitalization for respiratory infection	77/698 (11.0%)	116/1,231 (9.4%)	1.17	0.89	1.54	1.62	1.29	0.89	1.89	1.90	1.18	0.86	1.61	1.64
Hospitalization for gastrointestinal disease	23/698 (3.3%)	34/1,197 (2.8%)	1.19	0.71	2.01	1.67	1.13	0.56	2.31	1.51	1.32	0.71	2.48	1.97
Overweight or obesity at 5.5 years of age	50/506 (9.9%)	83/906 (9.2%)	1.08	0.77	1.51	1.37	1.05	0.68	1.62	1.28	1.00	0.69	1.45	1.00
Overweight or obesity at 9 years of age	56/450 (12.4%)	94/783 (12.0%)	1.04	0.76	1.41	1.24	0.83	0.52	1.32	1.70	0.86	0.60	1.25	1.60
*Developmental outcomes*														
Motor milestones not attained by the expected time (2.5 years)	43/641 (6.7%)	51/1,140 (4.5%)	1.50	1.01	2.22	2.37	0.86	0.50	1.48	1.60	1.14	0.72	1.80	1.54
Language milestones not attained by the expected time (2.5 years)	122/639 (19.1%)	172/1,140 (15.1%)	1.27	1.02	1.56	1.86	0.97	0.71	1.33	1.21	1.11	0.87	1.41	1.46
Cognitive milestones not attained by the expected time (5.5 years)	47/558 (8.4%)	81/998 (8.1%)	1.04	0.74	1.46	1.24	1.03	0.62	1.70	1.21	0.90	0.60	1.36	1.46
Social-emotional milestones not attained by the expected time (5.5 years)	84/557 (15.1%)	146/997 (14.6%)	1.03	0.80	1.32	1.21	0.90	0.66	1.24	1.46	0.98	0.74	1.29	1.16
Self-regulation problem (5.5 years)	73/557 (13.1%)	126/996 (12.7%)	1.04	0.79	1.36	1.24	1.02	0.73	1.43	1.16	0.99	0.73	1.33	1.11
Attention problem (8 years)	172/479 (35.9%)	328/870 (37.7%)	0.95	0.82	1.10	1.29	0.84	0.69	1.03	1.67	0.90	0.76	1.07	1.46
Adaptive problem (8 years)	186/470 (39.6%)	264/870 (33.2%)	1.30	1.12	1.52	1.92	1.27	1.04	1.55	1.86	1.27	1.08	1.50	1.86
Conduct problem (8 years)	166/475 (35.0%)	284/871 (32.6%)	1.07	0.92	1.25	1.34	1.03	0.84	1.27	1.21	1.04	0.87	1.24	1.24

RR: risk ratio, aRR: adjusted risk ratio, CI: confidence interval

^a^Adjusted for maternal age at delivery, multiple births, multiparity, fetal presentation, presence of fetal anomalies, maternal transport, pre-existing maternal medical conditions, pregnancy complications, maternal smoking during pregnancy, maternal alcohol consumption during pregnancy, maternal education attainment, paternal age at delivery, paternal education attainment, and place of residence at birth. In the obesity outcome model, maternal pre-pregnancy BMI (continuous) was also added as an adjustment variable.

**Fig. 2:**
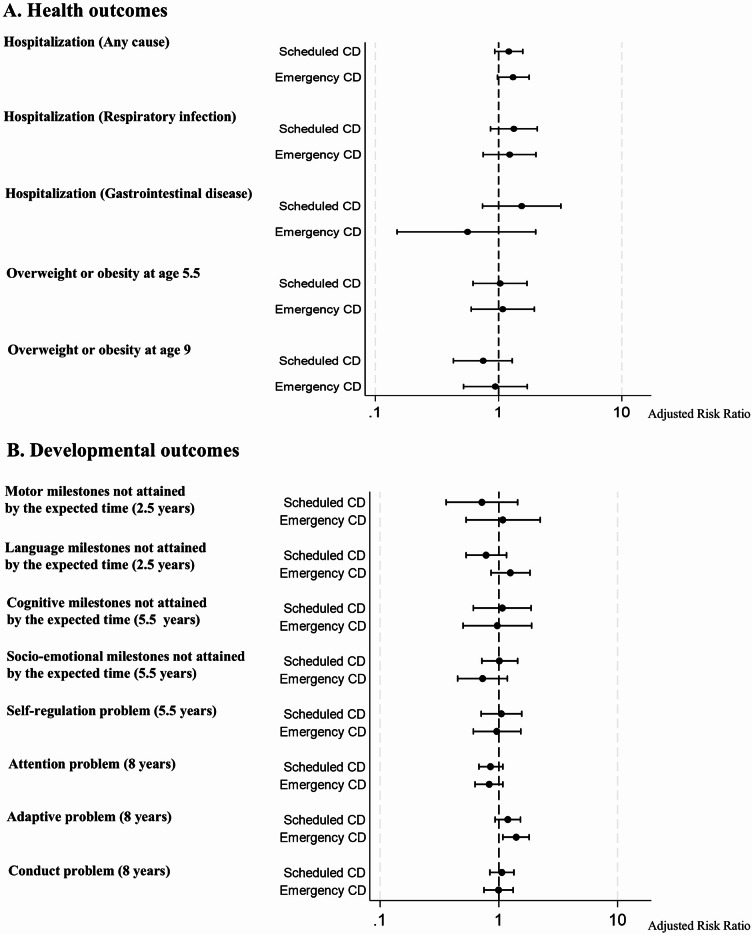
Adjusted risk ratios for multiple child health and developmental outcomes associated with cesarean section versus vaginal delivery, with 95% confidence intervals. Each outcome was analyzed separately. Adjusted for maternal age at delivery, parity, multiple births, prenatal fetal anomalies, presentation, maternal transfer status, maternal underlying diseases, pregnancy complications, maternal smoking during pregnancy, maternal alcohol consumption during pregnancy, maternal education attainment, paternal age, paternal education attainment, and place of residence at birth. Maternal pre-pregnancy body mass index was also adjusted in the obesity outcome model.

Supplementary analyses comparing scheduled and emergency CDs to vaginal delivery did not reveal statistically significant associations for most outcomes (Fig. 2). For hospitalization due to gastrointestinal disease, they showed slightly lower point estimate for risk with emergency CD (aRR 0.56, 95% CI 0.15–2.00) and higher point estimate for risk with scheduled CD (aRR 1.54, 95% CI 0.74–3.20), compared to vaginal delivery. For adaptive problems at 8 years, both types of CD showed slightly higher point estimates for risks compared to vaginal delivery, with emergency CD showing higher point estimates (aRR 1.40, 95%CI 1.08–1.80 for emergency CD; aRR 1.19, 95%CI 0.93–1.52 for scheduled CD). However, after Bonferroni correction for multiple comparisons, none of these associations remained statistically significant.

**Fig. 3:**
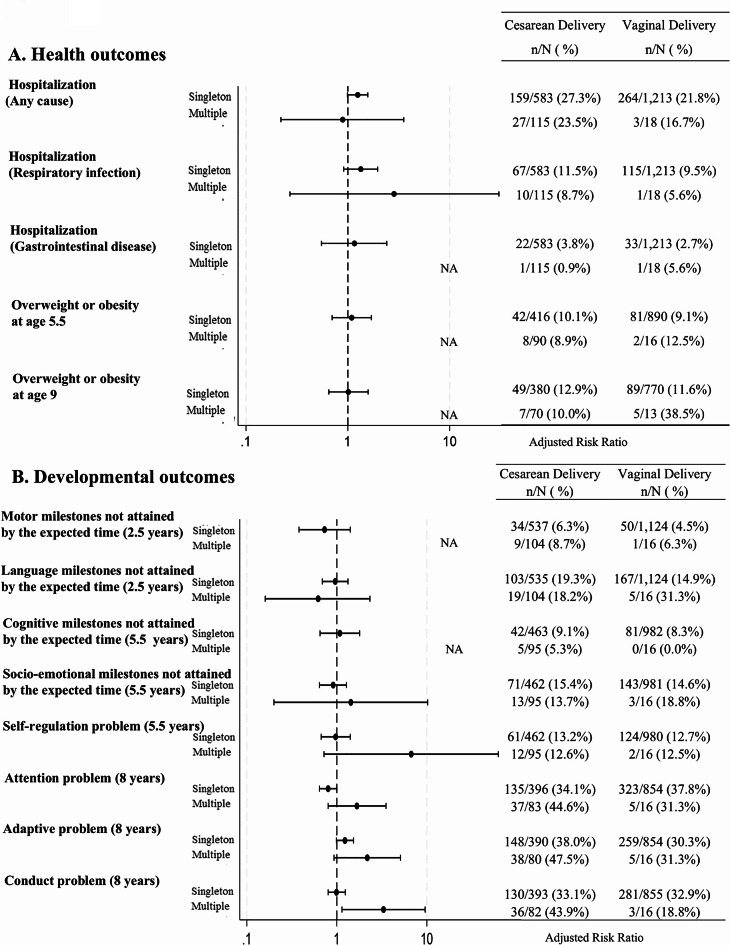
Adjusted risk ratios for multiple child health and developmental outcomes associated with cesarean section versus vaginal delivery, with 95% confidence intervals. Each outcome was analyzed separately. Adjusted for maternal age at delivery, parity, multiple births, prenatal fetal anomalies, presentation, maternal transfer status, maternal underlying diseases, pregnancy complications, maternal smoking during pregnancy, maternal alcohol consumption during pregnancy, maternal education attainment, paternal age, paternal education attainment, and place of residence at birth.

Subgroup analyses stratified by multiple births and preterm birth status had limited statistical power due to small sample sizes in these groups. In the multiple birth group (Fig. 3), the point estimates for the association between CD and several developmental outcomes appeared to be higher compared to the singleton group, although none of these differences reached statistical significance. These outcomes included self-regulation problems at 5.5 years, and attention, adaptive, and conduct problems at 8 years. Confidence intervals were wider in the multiple birth group, reflecting the smaller sample size and greater uncertainty in these estimates.

Analyses stratified by preterm birth status (Figure S1) showed different point estimates compared to those in the multiple birth stratified analysis. In the preterm group, CD was associated with lower point estimates for risks for certain outcomes, although none of these differences reached statistical significance. These outcomes included hospitalizations due to gastrointestinal disease and several developmental milestones (motor at 2.5 years, cognitive at 5.5 years, and attention and conduct problems at 8 years). Confidence intervals for these estimates were wider in the preterm group.

## Discussion

In this study based on data from a nationwide 2010 Japanese birth cohort, we examined the association between mode of delivery and several child health and developmental outcomes from 1.5 to 9 years of age. Our findings suggest that CD is not significantly associated with most child health and developmental outcomes, including hospitalizations, obesity, and various developmental milestones. While some non-significant differences were observed, the overall results do not provide strong evidence for long-term adverse effects of CD on child health and development in this cohort.

Our findings align with several previous studies reporting no significant long-term effects of CD on child health and development. However, they contrast with findings from the Japan Environment and Children’s Study (JECS)^22^, which suggested a potential association between CD and increased risk of autism spectrum disorder at age 3. This discrepancy may be attributed to several factors, including methodological differences, variations in outcome definitions, and differing follow-up periods. For instance, the JECS compared all births, potentially including a higher proportion of high-risk pregnancies in the CD group, whereas our study focused on births in hospitals managing high-risk deliveries. While this approach may limit generalizability, it potentially enhances the internal validity of our comparison between CD and vaginal delivery groups by reducing confounding from baseline risk factors. In addition to the different outcomes, our extended follow-up period to 8 years may have captured longer-term outcomes that differ from those observed at age 3 in the JECS.

Previous research on the long-term effects of CD on child outcomes worldwide has yielded inconsistent findings^33,39^. For example, while some studies have reported transient increases in BMI among infants born by CD^40^, others have found associations with increased obesity risk in older children^17^. These inconsistencies underscore the importance of considering country-specific or culture-specific factors when evaluating the impact of CD on long-term child health outcomes^41^. While mechanisms such as altered microbiome colonization due to bypassing exposure to maternal vaginal and intestinal flora during birth have been proposed to explain potential effects of CD^42^, our findings suggest that if such mechanisms are at play, their long-term impact may be minimal or effectively mitigated in the Japanese context. Japan’s healthcare system, characterized by universal health insurance coverage and standardized prenatal and postnatal care^43^, may contribute to optimizing outcomes regardless of delivery mode. For instance, Japan consistently reports one of the lowest perinatal mortality rates globally (2.8 per 1,000 total births in 2022)^44^. This combination may enable more accurate and timely identification of cases requiring CD, leading to optimal interventions that minimize risks associated with both underuse and overuse of the procedure. Furthermore, traditional dietary practices and overall child-rearing approaches in Japan may contribute to offsetting potential negative effects of CD on infant health and development, including impacts on the microbiome^45^. Collectively, these healthcare and cultural factors create an environment where the potential risks of CD are minimized, possibly explaining the lack of significant long-term negative outcomes observed in our study.

Supplementary analyses comparing scheduled and emergency CDs to vaginal delivery did not reveal statistically significant associations for most outcomes. This nuanced analysis provides additional reassurance about the safety of both types of CD when medically indicated. However, some non-significant differences were observed, such as a higher point estimates for the risk of adaptive problems at 8 years for both types of CD, which warrant further investigation in larger studies.

Subgroup analyses stratified by multiple births and preterm birth status were limited by small sample sizes, resulting in wide confidence intervals. These analyses revealed some non-significant differences: higher point estimates for the association between CD and certain developmental outcomes in multiple births, while lower point estimates were found for the association between CD and these outcomes in the preterm group. These observations are consistent with previous research hypotheses suggesting that CD might offer benefits for preterm infants. Lower point estimates for the associations between CD and certain outcomes in preterm infants could be attributed to CD’s potential to mitigate peripartum stress and complications often associated with preterm vaginal delivery. Moreover, Japan’s high quality prenatal and postnatal care system may contribute to optimizing selection of delivery mode and timing. However, these findings should be considered highly preliminary due to smaller sample size and lack of statistical significance and require confirmation in larger studies.

The strengths of our study include its use of a large, nationally representative birth cohort, the linkage to the comprehensive Perinatal Research Network database, and the wide range of outcomes examined over an extended period. Our outcome-wide approach allowed for a holistic assessment of CD’s potential impacts, reducing the risk of selective reporting bias. The inclusion of both scheduled and emergency CD in our supplementary analyses provided insights into potential differences between these delivery methods. However, our study also has limitations. First, the relatively small sample size (*n* = 2,116) and inclusion of only Japanese children may limit generalizability, particularly as the linked perinatal database tends to oversample high-risk pregnancies. However, key outcome proportions in our sample were not markedly different from those in the original cohort, which represents all births in Japan (Table S4). Second, potential selection bias due to loss to follow-up may have affected our results, as participants lost to follow-up were more likely to have mothers with lower educational attainment and a history of smoking. However, the proportion of loss to follow-up was similar between CD and vaginal delivery groups, suggesting that impact of attrition bias on our main findings may be limited. Third, child development was assessed using binary responses to survey items without validated scales, potentially limiting the capture of nuanced developmental variations. Fourth, while we calculated E-values to assess the potential impact of unmeasured confounding, residual confounding from factors such as family history of neurodevelopmental disorders cannot be ruled out. Fifth, our study had several limitations regarding the factors that may influence delivery method selection. While prenatal imaging plays a crucial role in determining delivery methods, we did not examine its potential effects on fetal health or delivery outcomes. Additionally, our study design did not allow for comprehensive analysis of maternal physiological conditions during pregnancy or regional environmental factors that could affect delivery decisions. These unexplored variables, including but not limited to maternal vaginal microbiome changes during pregnancy and local climate conditions, represent important areas for future research to better understand their impact on delivery method selection and outcomes. Lastly, the sample size for some subgroup analyses was limited, and effects beyond 9 years of age remain unknown.

## Conclusions

Our findings suggest that CD does not significantly impact most long-term adverse child health and developmental outcomes in this Japanese cohort up to 9 years of age. This provides cautious reassurance to parents and healthcare providers about the long-term safety of CD when medically indicated. However, it is important to note that the absence of statistically significant associations does not necessarily imply the absence of clinically relevant effects. Future studies should consider longer-term follow-up, larger sample sizes for subgroup analyses, and more detailed assessment of potential mediating factors such as microbiome development. As medical practices continue to evolve, ongoing research will be crucial to ensure the optimal balance between necessary medical interventions and potential long-term health outcomes, particularly in high-risk populations such as preterm infants and multiple births.

## Electronic supplementary material

Below is the link to the electronic supplementary material.

Supplementary Material 1

## Data Availability

De-identified individual participant data will be made available upon reasonable request and approval by the Ministry of Health, Labour and Welfare and the Japan Society of Obstetrics and Gynecology. For data requests, please contact the corresponding author (naomim@okayama-u.ac.jp).

## Declarations

**Declaration of generative AI and AI-assisted technologies in the writing process**

During the preparation of this work the authors used an AI-powered language model (Claude) in order to assist with English language editing and proofreading. After using this tool/service, the authors reviewed and edited the content as needed and take full responsibility for the content of the publication.
