# Supplementary material for: Cesarean delivery on child health and development in Japanese nationwide birth cohort
Source: Sci Rep. 2025 Jan 20;15:2485. doi: 10.1038/s41598-025-87043-2 (PMC11747486; doi:10.1038/s41598-025-87043-2)
Supplement: Supplementary file 1 — Supplementary Material 1 [file 41598_2025_87043_MOESM1_ESM.pdf]

Supplementary materials

Cesarean Delivery and Child Health and Development: Nationwide birth cohort in Japan

Naomi Matsumoto*, MD, PhD[1]; Takashi Mitsui, MD, PhD[2]; Kei Tamai, MD, PhD[3]; Tomoya Hirota, MD, PhD[4]; Hisashi Masuyama, MD, PhD[2]; Takashi Yorifuji, MD, PhD[1]

Affiliations:

[1] Department of Epidemiology, Faculty of Medicine, Dentistry and Pharmaceutical Sciences, Okayama University, Okayama, Japan

[2] Department of Obstetrics and Gynecology, Okayama University Graduate School of Medicine, Okayama, Japan

[3]Division of Neonatology, NHO Okayama Medical Center, Okayama, Japan

[4] Department of Psychiatry and Behavioral Sciences, UCSF Weill Institute for Neurosciences, University of California San Francisco, San Francisco

eMethod. Descriptions of Exposure and Outcomes

Table S1. Questionnaires for Assessing Developmental Milestones and Behavioral Indicators

Table S2. Characteristics of Participants Who Were Lost to Follow-up

Table S3. The detailed numbers and percentages of the evaluated outcomes

Figure S1. Adjusted risk ratios for preterm child health and developmental outcomes associated with cesarean section versus vaginal delivery, with 95% confidence intervals.

Table S4. Proportions of Each Outcome among Children Analyzed and All Children with mothers aged over 25 years included in 2010 Cohort

eMethod. Descriptions of Exposure and Outcomes

This section provides comprehensive definitions and measurement methods for the primary exposure (mode of delivery) and all outcomes examined in this study, including hospitalizations, metabolic status, and developmental milestones.

Information on delivery methods

The PRN database categorizes delivery methods into six choices: “natural vaginal,” “suction,” “forceps,” “scheduled cesarean,” “emergency cesarean,” and “other.” Of these, those who selected “scheduled cesarean” or “emergency cesarean” were defined as the cesarean section group, whereas those who chose “natural vaginal,” “suction,” or “forceps” were classified as the vaginal delivery group.

Information on child health outcomes

Preschool hospitalizations

In the Longitudinal Survey of Babies in the 21st Century, parents were queried annually about whether their children had been admitted to the hospital for treatment in the previous year and the reason for admission. The preschool hospitalization group consisted of children who experienced at least one admission for any reason between the ages of 1.5 and 5.5 years based on information from the second through sixth surveys. Additionally, we defined the preschool respiratory infection hospitalization group as consisting of children who were hospitalized at least once during the same period for respiratory tract infections. We defined the preschool gastrointestinal tract illness hospitalization group as children who were hospitalized at least once during the same period for gastroenteritis or other gastrointestinal tract illnesses excluding intestinal intussusception. The

frequency of hospitalization in each period was not assessed in this survey.

Metabolic status

BMI standard deviation scores were computed using anthropometric data collected at 5.5 and 9 years of age in accordance with the World Health Organization's established criteria. The participants were stratified into the following categories based on their BMI standard deviation scores: underweight (< 1.5), normal weight (−1.5 to 1.5), overweight (1.5 to 2.5), and obese (≥ 2.5). To minimize the potential impact of outliers, the children who exhibited BMI standard deviation scores less than −5 at 5.5 years (n = 4, comprising three vaginal deliveries and one cesarean section) and 9 years (n = 1, one vaginal delivery) were excluded from the analysis of weight status. For this investigation, the overweight and obese categories were consolidated into a single obese group.

Developmental milestones

The Longitudinal Survey of Babies in the 21st Century incorporates a comprehensive assessment of child development across multiple domains and time points. The responses for all of the child development items are dichotomized (“Yes” and “No”). Two board-certified experts, a pediatrician (NM) and a child psychiatrist specializing in child development (TH), meticulously evaluated and classified each survey item into the following developmental domains (Table S1): motor milestones (4 items) at age 2.5 years, language milestones 3 items) at age 2.5 years, cognitive milestones 1 item) at age 5.5 years, self-regulation 2 items) at age 5.5 years, social-emotional milestones 3 items) at age 5.5 years, attention (scale: 1 item) at age 8 years, adaptive skills (3 items) at age 8 years, and conduct problems 3 items) at age 8 years. Failure to achieve any single item within a domain was considered as not having achieved development in that domain.

The delineation of these developmental domains was established through a consensus between the aforementioned experts and drew upon milestone assessments from the Centers for Disease Control and Prevention; the Behavioral Assessment System for Children, Third Edition; and the Strengths and Difficulties Questionnaire. The operational definitions of the domains and their constituent items were subsequently reviewed and approved by an additional pediatric physician (TY).

Comprehensive details regarding the specific assessment items and the computational methods that were employed for the milestones and scales are provided in Table S1.

Table S1. Questionnaires for Assessing Developmental Milestones and Behavioral Indicators

Questionnaires	Developmental milestones	Reference
The following items were selected as "yes" or "no" in the survey at 2.5 years of age		
Can your child walk?	Motor milestone	CDC
Can your child run?	Motor milestone	CDC
Can your child climb stairs?	Motor milestone	CDC
Can your child use a spoon (fork) for self-feeding?	Motor milestone	CDC
Can your child say words with meaning?	Language milestone	Expert opinion referred to CDC
Can your child compose two-phrase sentences?	Language milestone	CDC
Can your child say his or her own name?	Language milestone	CDC
The following items were selected as "can" or "cannot" in the survey at 5.5 years of age		
Focusing on one thing	Cognitive milestones	CDC
Expressing emotions appropriately	Social-emotional milestones	Expert opinion
Acting in a group	Social-emotional milestones	Expert opinion
Keeping promises	Social-emotional milestones	CDC
Listening calmly	Self-regulation	Expert opinion
Enduring or tolerating	Self-regulation	Expert opinion
The following behavioral indicators were selected as "has acquired" or "has not acquired" in the survey at 8 years of age		
Listens to the end of what others say	Attention problem	BASC3 (PRS)
Greets and responds to others	Adaptive skills	BASC3 (PRS)
Always looks right and left when crossing the road	Adaptive skills	BASC3 (PRS)
Does not follow strangers	Adaptive skills	BASC3 (PRS)
Does not tell a lie	Conduct problem	SDQ_18/BASC3 (PRS)
Does not break toys or books	Conduct problem	BASC3/CBCL
Is not violent to others	Conduct problem	SDQ_18/BASC3 (PRS)

CDC: Centers for Disease Control and Prevention,

BASC3 (PRS): Behavior Assessment System for Children, Third Edition, Parent Rating Scales,

SDQ: Strengths and Difficulties Questionnaire,

CBCL: Child Behavior Checklist.

Table S2. Characteristics of Participants Who Were Lost to Follow-up

	Analyzed (n=1,233)	Lost to follow up (n=894)	Total (N=2,114)
Mode of delivery			
Vaginal delivery	783 (63.5%)	568 (64.5%)	1,351 (63.9%)
Cesarean section	450 (36.5%)	313 (35.5%)	763 (36.1%)
Gestational week*	38.05 (2.48)	37.83 (2.67)	37.96 (2.56)
Birth weight (g)*	2821.48 (562.77)	2809.09 (605.18)	2816.32 (580.71)
Low birth weight < 2500g	269 (21.8%)	202 (22.9%)	471 (22.3%)
Small for gestational age	70 (5.7%)	51 (5.8%)	121 (5.7%)
Multiple births	83 (6.7%)	66 (7.5%)	149 (7.0%)
Neonatal asphyxia			
No	1,188 (97.0%)	846 (96.6%)	2,034 (96.8%)
Mild neonatal asphyxia	34 (2.8%)	25 (2.9%)	59 (2.8%)
Severe neonatal asphyxia	3 (0.2%)	5 (0.6%)	8 (0.4%)
Maternal transfer			
No	1,052 (85.3%)	732 (83.1%)	1,784 (84.4%)
Yes (emergent)	73 (5.9%)	61 (6.9%)	134 (6.3%)
Yes (non-urgent)	108 (8.8%)	88 (10.0%)	196 (9.3%)
Parity			
Primipara	654 (53.3%)	458 (52.5%)	1,112 (53.0%)
Multipara	573 (46.7%)	415 (47.5%)	988 (47.0%)
Maternal underlying conditions	392 (31.8%)	268 (30.4%)	660 (31.2%)
in-vitro fertilization	79 (6.4%)	37 (4.2%)	116 (5.5%)
Position			
Cephalic presentation	1,134 (92.0%)	821 (93.2%)	1,955 (92.5%)
Breech presentation	99 (8.0%)	60 (6.8%)	159 (7.5%)
Pregnancy complications	731 (59.3%)	499 (56.6%)	1,230 (58.2%)
Prenatal fetal anomalies	99 (8.0%)	64 (7.3%)	163 (7.7%)
Maternal smoking during pregnancy	24 (2.6%)	57 (8.8%)	81 (5.1%)
Maternal alcohol consumption during pregnancy	35 (3.8%)	26 (4.0%)	61 (3.9%)
Maternal age at delivery			
<30	295 (23.9%)	339 (38.5%)	634 (30.0%)
30~34	448 (36.3%)	302 (34.3%)	750 (35.5%)
35~	490 (39.7%)	240 (27.2%)	730 (34.5%)
Paternal age at delivery			
<30	227 (18.6%)	246 (29.3%)	473 (22.9%)
30~34	372 (30.4%)	253 (30.2%)	625 (30.3%)
35~	624 (51.0%)	340 (40.5%)	964 (46.8%)
Maternal education attainment			
Bachelor's degree or higher	399 (33.3%)	166 (25.6%)	565 (30.6%)
Vocational school/junior college graduate	523 (43.6%)	268 (41.4%)	791 (42.8%)
High school graduate or below	277 (23.1%)	214 (33.0%)	491 (26.6%)
Paternal education attainment			
Bachelor's degree or higher	686 (57.7%)	284 (45.2%)	970 (53.4%)
Vocational school/junior college graduate	178 (15.0%)	111 (17.7%)	289 (15.9%)
High school graduate or below	325 (27.3%)	233 (37.1%)	558 (30.7%)
Place of residence at birth			
Special ward or designated city	532 (43.1%)	376 (42.7%)	908 (43.0%)
City	638 (51.7%)	443 (50.3%)	1,081 (51.1%)
Town or village	63 (5.1%)	62 (7.0%)	125 (5.9%)

Categorical variables were described by number (%), and continuous variables* were described by mean (standardized deviation).

Table S3. The detailed numbers and percentages of the evaluated outcomes

	Delivery method		
	Vaginal delivery (n=1,351)	Cesarean section (n=763)	All (N=2,114)
Hospitalization for any reason			
No	964 (78.3%)	512 (73.4%)	1,476 (76.5%)
Yes	267 (21.7%)	186 (26.6%)	453 (23.5%)
Hospitalization for respiratory infection			
No	1,115 (90.6%)	621 (89.0%)	1,736 (90.0%)
Yes	116 (9.4%)	77 (11.0%)	193 (10.0%)
Hospitalization for gastrointestinal disease			
No	1,197 (97.2%)	675 (96.7%)	1,872 (97.0%)
Yes	34 (2.8%)	23 (3.3%)	57 (3.0%)
Overweight or obesity (5.5 years)			
No	823 (90.8%)	456 (90.1%)	1,279 (90.6%)
Yes	83 (9.2%)	50 (9.9%)	133 (9.4%)
Overweight or obesity (9 years)			
No	689 (88.0%)	394 (87.6%)	1,083 (87.8%)
Yes	94 (12.0%)	56 (12.4%)	150 (12.2%)
Motor milestones not attained by the expected time (2.5 years)			
0	1,089 (95.5%)	598 (93.3%)	1,687 (94.7%)
1	31 (2.7%)	29 (4.5%)	60 (3.4%)
2	6 (0.5%)	2 (0.3%)	8 (0.4%)
3	6 (0.5%)	6 (0.9%)	12 (0.7%)
4	8 (0.7%)	6 (0.9%)	14 (0.8%)
Language milestones not attained by the expected time (2.5 years)			
0	968 (84.9%)	517 (80.9%)	1,485 (83.5%)
1	88 (7.7%)	77 (12.1%)	165 (9.3%)
2	64 (5.6%)	29 (4.5%)	93 (5.2%)
3	20 (1.8%)	16 (2.5%)	36 (2.0%)
Cognitive milestones not attained by the expected time (5.5 years)			
0	917 (91.9%)	511 (91.6%)	1,428 (91.8%)
1	81 (8.1%)	47 (8.4%)	128 (8.2%)
Self-regulation problem (5.5 years)			
0	870 (87.3%)	484 (86.9%)	1,354 (87.2%)
1	98 (9.8%)	57 (10.2%)	155 (10.0%)
2	28 (2.8%)	16 (2.9%)	44 (2.8%)
Social-emotional milestones not attained by the expected time (5.5 years)			
0	851 (85.4%)	473 (84.9%)	1,324 (85.2%)
1	106 (10.6%)	61 (11.0%)	167 (10.7%)
2	25 (2.5%)	13 (2.3%)	38 (2.4%)
3	15 (1.5%)	10 (1.8%)	25 (1.6%)
Attention problem (8 years)			
0	721 (82.0%)	351 (73.6%)	1,072 (79.1%)
1	158 (18.0%)	126 (26.4%)	284 (20.9%)
Adaptive problem (8 years)			
0	606 (69.7%)	284 (60.4%)	890 (66.4%)
1	202 (23.2%)	155 (33.0%)	357 (26.6%)
2	55 (6.3%)	25 (5.3%)	80 (6.0%)
3	7 (0.8%)	6 (1.3%)	13 (1.0%)
Conduct problem (8 years)			
0	587 (67.4%)	309 (65.1%)	896 (66.6%)
1	206 (23.7%)	109 (22.9%)	315 (23.4%)
2	59 (6.8%)	45 (9.5%)	104 (7.7%)
3	19 (2.2%)	12 (2.5%)	31 (2.3%)

For detailed definitions of each developmental milestone, please refer to Table S1.

Figure S1.

A. Health outcomes

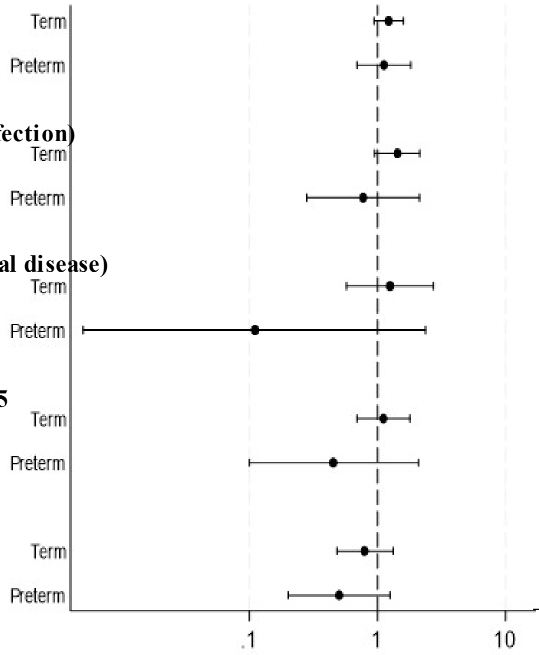

Outcome		Cesarean Delivery n/N (%)	Vaginal Delivery n/N (%)
**Hospitalization (Any cause)**	Term	128/530 (24.2%)	236/1,123 (21.0%)
	Preterm	58/168 (34.5%)	31/108 (28.7%)
**Hospitalization (Respiratory infection)**	Term	55/530 (10.4%)	100/1,123 (8.9%)
	Preterm	22/168 (13.1%)	16/108 (14.8%)
**Hospitalization (Gastrointestinal disease)**	Term	20/530 (3.8%)	31/1,123 (2.8%)
	Preterm	3/168 (1.8%)	3/108 (2.8%)
**Overweight or obesity at age 5.5**	Term	39/388 (10.1%)	77/823 (9.4%)
	Preterm	11/118 (9.3%)	6/83 (7.2%)
**Overweight or obesity at age 9**	Term	42/346 (12.1%)	83/715 (11.6%)
	Preterm	14/104 (13.5%)	11/68 (16.2%)

Adjusted Risk Ratio

B. Developmental outcomes

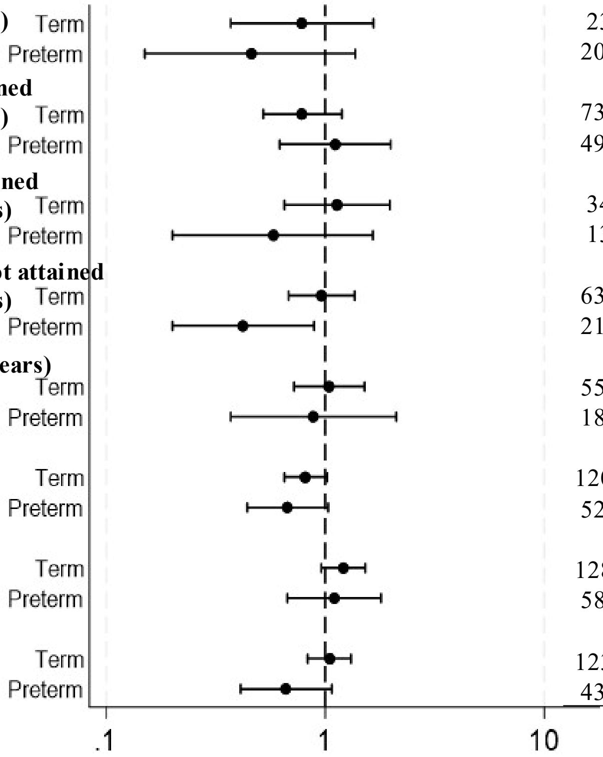

Outcome		Cesarean Delivery n/N (%)	Vaginal Delivery n/N (%)
**Motor milestones not attained by the expected time (2.5 years)**	Term	23/488 (4.7%)	41/1,042 (3.9%)
	Preterm	20/153 (13.1%)	10/98 (10.2%)
**Language milestones not attained by the expected time (2.5 years)**	Term	73/487 (15.0%)	142/1,041 (13.6%)
	Preterm	49/152 (32.2%)	30/99 (30.3%)
**Cognitive milestones not attained by the expected time (5.5 years)**	Term	34/427 (8.0%)	67/908 (7.4%)
	Preterm	13/131 (9.9%)	14/90 (15.6%)
**Socio-emotional milestones not attained by the expected time (5.5 years)**	Term	63/426 (14.8%)	124/907 (13.7%)
	Preterm	21/131 (16.0%)	22/90 (24.4%)
**Self-regulation problem (5.5 years)**	Term	55/426 (12.9%)	108/906 (11.9%)
	Preterm	18/131 (13.7%)	18/90 (20.0%)
**Attention problem (8 years)**	Term	120/358 (33.5%)	295/795 (37.1%)
	Preterm	52/121 (43.0%)	33/75 (44.0%)
**Adaptive problem (8 years)**	Term	128/352 (36.4%)	236/795 (30.0%)
	Preterm	58/118 (49.2%)	28/75 (37.3%)
**Conduct problem (8 years)**	Term	123/357 (34.5%)	255/796 (32.0%)
	Preterm	43/118 (36.4%)	29/75 (38.7%)

Adjusted Risk Ratio

Figure S1. Adjusted risk ratios for multiple child health and developmental outcomes associated with cesarean section versus vaginal delivery, stratified by preterm birth status, with 95% confidence intervals. Each outcome was analyzed separately. Adjusted for maternal age at delivery, parity, multiple births, prenatal fetal anomalies, presentation, maternal transfer status, maternal underlying diseases, pregnancy complications, maternal smoking during pregnancy, maternal alcohol consumption during pregnancy, maternal education attainment, paternal age, paternal education attainment, and place of residence at birth.

Table S4. Proportions of Each Outcome among Children Analyzed and All Children with mothers aged over 25 years included in 2010 Cohort

			Analyzed		Original 2010 Cohort		
Hospitalization for any reason	Yes		23.5	%	21.2	%	
Hospitalization for respiratory infection	Yes		10.0	%	8.8	%	
Hospitalization for gastrointestinal disease	Yes		3.0	%	2.9	%	
Overweight or obesity at age 5.5 years	Yes		9.4	%	10.0	%	
Overweight or obesity at age 9 years	Yes		12.2	%	13.1	%	
Motor milestones not attained by the expected time (2.5 years)	Mean (SD)		0.09	(0.48)	0.05		(0.30)
		1	3.4	%	2.5	%	
	Score	2	0.5	%	0.3	%	
		3	0.7	%	0.2	%	
		4	0.8	%	0.2	%	
Language milestones not attained by the expected time (2.5 years)	Mean (SD)		0.26	(0.65)	0.16		(0.49)
		1	9.3	%	8.0	%	
	Score	2	5.2	%	3.1	%	
		3	2.0	%	0.7	%	
Cognitive milestones not attained by the expected time (5.5 years)	Mean (SD)		0.08	(0.27)	0.07		(0.26)
	Score	1	8.2	%	7.2	%	
Social-emotional milestones not attained by the expected time (5.5 years)	Mean (SD)		0.2	(0.56)	0.17		(0.49)
		1	10.8	%	9.7	%	
	Score	2	2.5	%	2.4	%	
		3	1.6	%	0.9	%	
Self-regulation problem (5.5 years)	Mean (SD)		0.16	(0.43)	0.14		(0.40)
	Score	1	10.0	%	10.2	%	
		2	2.8	%	1.9	%	
Attention problem (8 years)	Mean (SD)		0.21	(0.41)	0.19		(0.39)
	Score	1	20.9	%	18.8	%	
Adaptive problem (8 years)	Mean (SD)		0.41	(0.65)	0.38		(0.63)
		1	26.6	%	23.7	%	
	Score	2	6.0	%	5.8	%	
		3	1.0	%	0.8	%	
Conduct problem (8 years)	Mean (SD)		0.46	(0.74)	0.43		(0.69)
		1	23.4	%	23.9	%	
	Score	2	7.7	%	7.0	%	
		3	2.3	%	1.5	%	

SD: standardized deviation
